# The complication sum score: A Delphi-based approach to summarize treatment related complications for esophageal cancer

**DOI:** 10.1016/j.ctro.2026.101146

**Published:** 2026-03-15

**Authors:** M.L. Frederiks, M. Berbée, B.P.L. Wijnhoven, E. Schuit, P.S.N. van Rossum, G.J. Meijer, S. Mook, J.J. Nuyttens, H. Rütten, M.D. den Hartogh, B. van Etten, J.A. Langendijk, H.W.M. van Laarhoven, C.T. Muijs, MODELS consortium, C.T. Muijs, B.P.L. Wijnhoven, H.W.M. van Laarhoven, M. Berbee, J.A. Langendijk, H.P. van der Laan, A. van der Schaaf, J.J.M.E. Nuyttens, G.J. Meijer, H. Rutten, Y.L.B. Klaver, M.L. Frederiks, R.H.A. Verhoeven, E. Schuit, B. van Etten, J.J. de Haan, W. Kleder, M.I. van Berge Henegouwen, P.S.N. van Rossum, Z. van Kesteren, B. Mostert, W. Schillemans, M. Sosef, F.A.R.M. Warmerdam, R. Canters, J.P. Ruurda, S. Mook, N. Haj Mohammad, B. Klarenbeek, M.D. den Hartogh, H. Westdorp

**Affiliations:** n Department of Radiation Oncology, University of Groningen, University Medical Center Groningen, Groningen, the Netherlands; o Department of Surgery, Erasmus MC Cancer Institute University Medical Center, Rotterdam, the Netherlands; p Department of Medical Oncology, Amsterdam UMC, location University of Amsterdam, Meibergdreef 9, 1105 AZ Amsterdam, the Netherlands; q Cancer Centre Amsterdam, Imaging and Biomarkers, Amsterdam, the Netherlands; r Department of Radiation Oncology (MAASTRO), Maastro Clinic, Maastricht, the Netherlands; s Department of Radiation Oncology, Erasmus MC Cancer Institute University Medical Center, Rotterdam, the Netherlands; t Department of Radiation Oncology, University Medical Center Utrecht, Utrecht University, Utrecht, the Netherlands; u Department of Radiation Oncology, Radboud University Nijmegen Medical Centre, Nijmegen, the Netherlands; v HollandPTC, Delft, the Netherlands; w Department of Radiation Oncology, Leiden University Medical Center, Leiden, the Netherlands; x Netherlands Comprehensive Cancer Organisation (IKNL), Department of Research & Development, Utrecht, the Netherlands; y Amsterdam UMC location University of Amsterdam, Department of Medical Oncology, Amsterdam, the Netherlands; z Cancer Center Amsterdam, Cancer Treatment and Quality of Life, Amsterdam, the Netherlands; aa Development, Netherlands Comprehensive Cancer Organisation, Utrecht, the Netherlands; ab Department of Epidemiology & Health Economics, Julius Center for Health Sciences and Primary Care, University Medical Center Utrecht, Utrecht University, Utrecht, the Netherlands; ac Department of Surgery, University Medical Center Groningen, Groningen, the Netherlands; ad Department of Medical Oncology, University of Groningen, University Medical Center Groningen, Groningen, the Netherlands; ae Department of Surgery, Martini Hospital, Groningen, the Netherlands; af Department of Surgery, Amsterdam UMC, De Boelelaan 1017, Amsterdam 1081HV, the Netherlands; ag Department of Radiation Oncology, Amsterdam UMC Location VUmc, De Boelelaan 1017, Amsterdam 1081HV, the Netherlands; ah Department of Radiation Oncology, Amsterdam UMC, De Boelelaan 1017, Amsterdam 1081HV, the Netherlands; ai Department of Medical Oncology, Erasmus MC Cancer Institute University Medical Center, Rotterdam, the Netherlands; aj Department of Surgery, Zuyderland Medical Center, Heerlen, the Netherlands; ak Department of Medical Oncology, Zuyderland Medical Center, Sittard-Geleen, the Netherlands; al Department of Surgery, University Medical Center Utrecht, Utrecht University, Utrecht, the Netherlands; am Department of Radiotherapy, University Medical Center Utrecht, Utrecht University, Utrecht, the Netherlands; an Department of Medical Oncology, University Medical Center Utrecht, Utrecht University, Utrecht, the Netherlands; ao Department of Surgery, Radboud University Nijmegen Medical Centre, Nijmegen, the Netherlands; ap Radiotherapiegroep, Arnhem/Deventer, the Netherlands; aq Department of Medical Oncology, Radboud University Nijmegen Medical Centre, Nijmegen, the Netherlands; a Department of Radiation Oncology, University Medical Center Groningen, Groningen, the Netherlands; b Department of Radiation Oncology (MAASTRO), Maastro Clinic, Maastricht, the Netherlands; c Department of Surgery, Erasmus MC Cancer Institute, Erasmus University Medical Centre, Rotterdam, the Netherlands; d Department of Epidemiology & Health Economics, Julius Center for Health Sciences and Primary Care, University Medical Center Utrecht, Utrecht University, Utrecht, the Netherlands; e Department of Radiation Oncology, Amsterdam UMC (VUmc Amsterdam), the Netherlands; f Cancer Center Amsterdam, Imaging and Biomarkers, Amsterdam, the Netherlands; g Department of Radiation Oncology, University Medical Center Utrecht, Utrecht, the Netherlands; h Department of Radiation Oncology, Erasmus University Medical Centre, Rotterdam, the Netherlands; i Department of Radiation Oncology, Radboud University Nijmegen Medical Centre, Nijmegen, the Netherlands; j Radiotherapiegroep, Arnhem/Deventer, the Netherlands; k Department of Surgery, University Medical Center Groningen, Groningen, the Netherlands; l Amsterdam UMC, location University of Amsterdam, Department of Medical Oncology, De Boelelaan 1017, 1081 HV Amsterdam, the Netherlands

**Keywords:** Neoadjuvant chemoradiotherapy, Treatment-related toxicity, Delphi consensus, Normal tissue complication probability, Esophageal cancer

## Abstract

**Background and purpose:**

Prevention of treatment-related complications remains a major challenge in esophageal cancer (EC) patients undergoing neoadjuvant chemoradiotherapy (nCRT) ± surgery. A single measure, reflecting the total complication burden, may provide a clear overall picture of patient burden and enables a direct comparison of alternative treatment strategies. We developed a Complication Sum Score (CSS) to quantify symptomatic complications.

**Materials and methods:**

A Delphi consensus procedure was used to develop the CCS using the expertise of health professionals to assess the impact of a spectrum of complications. The impact of each complication was scored on a 0–100 scale. Consensus for these scores was defined as an interquartile range ≤ 20. The association between the CSS and hospital/ICU stay, overall survival (OS) at 1 year and quality of life (QoL) was assessed in a multicenter dataset of patients with EC, using multivariable (logistic) regression analysis, correcting for possible confounders.

**Results:**

Forty-five experts completed the two-round Delphi procedure. Consensus was reached for all (36) complications. In 1225 patients, that underwent nCRT and esophagectomy, a higher CSS was significantly associated with a longer hospital (coefficient = 0.43[0.38–0.48], p < 0.01) and ICU stay (coefficient = 0.23[0.19–0.26], p < 0.01), and a worse OS (odds ratio = 1.005[1.004–1.006], p < 0.01). Among the patients that completed a QoL questionnaire after surgery (n = 98) an increased CSS was associated with worse QoL (coefficient = -0.24[-0.01–0.49], p = 0.06).

**Conclusion:**

The CSS offers a comprehensive tool to quantify treatment-related complications in a single score in EC patients that receive nCRT followed by surgical resection.

## Highlights

• Complication Sum Score measures oesophageal cancer treatment burden.
• Delphi consensus among 45 European experts weighed 36 key complications.
• Higher Complication Sum Scores link to longer hospital and ICU stays.
• Higher Complication Sum Scores link to reduced 1-year survival.
• Higher Complication Sum Scores trend to worse quality of life.

## Introduction

Treatment-related complications remain a significant challenge in curative treatment of patients with esophageal cancer (EC) [1], [2]. Neo-adjuvant chemoradiotherapy (nCRT) appears to contribute to the etiology of some of these complications, as they are associated with radiation dose to organs at risk (OARs). For example, cardiac and immunological effects have been linked to dose to the heart, while pulmonary complications are associated with dose to the lungs [3], [4], [5].

As such, the quality of the radiotherapy is essential for an effective treatment. Lowering the radiation dose to relevant OARs could reduce the risk of complications, even post-operative complications. However, reducing the dose to one OAR often results in an increased dose to other OARs and it remains an unresolved challenge how to balance the dose exposures to several OARs for each patient.

Currently, endpoint-directed optimization [6], i.e. treatment planning using multiple normal tissue complication probability (NTCP) models, is one of the most promising approaches to balance OAR doses. However, this requires NTCP models for each relevant complication. Furthermore, these complications should be weighed against each other regarding their clinical impact on patients. As a solution, Van der Laan et al. [7] integrated various NTCP models into a toxicity profile, considering the predicted impact of complications on quality of life (QoL). However, this approach is difficult in EC patients, as QoL assessments in the early post-operative phase, particularly during ICU-admission, is rarely possible due to the condition of the patient [8].

A single burden score could be an alternative to quantify the overall impact for patients, as it combines various treatment-related complications. Existing scores, such as the total toxicity burden (TTB) [9] and comprehensive complication index (CCI) [10], address this challenge but have limitations. The TTB includes asymptomatic complications (of limited clinical relevance), and the CCI focuses solely on postoperative complications and does not consider other treatment-related complications.

To address these gaps, we aim to develop the Complication Sum Score (CSS), a novel metric that aggregates symptomatic complications which can not only be attributed to the surgery, but are also potentially associated to radiation dose to the OARs in EC patients undergoing trimodality treatment. Unlike the CCI, which focuses exclusively on postoperative complications, and the TTB, which includes asymptomatic events, the CSS integrates all symptomatic complications potentially linked to radiotherapy. Additionally, we investigated the association of the newly developed CSS with relevant outcome measures including hospital and ICU stay, overall survival, and quality of life. We hypothesize that the CSS will serve as a tool to quantify the treatment burden for a patient by combining several severity-weighted symptomatic complications into one score.

## Methods

The CSS was developed via a modified Delphi consensus and evaluated for its associations with various clinical outcomes. The Delphi study was conducted using an online survey platform (RedCap [11], [12]). The clinical associations of the CSS were assessed using the MODELS cohort (clinicaltrials.gov: NCT06366828), a database of EC patients treated between 2015 and 2021. The study protocol received approval from the medical ethics committee of the University Medical Centre Groningen (11446).

### Complication selection

The initial list of complications was based on two prospective data registries for EC patients in the Netherlands: the Netherlands Cancer Registry (IKNL) and the Dutch Upper GI Cancer Audit (DUCA) [13], [14]. In these data registries 55 complications were identified. Complications were excluded if ≥ 50% of an advisory panel (three radiation oncologists (M.B., J.L., and C.M.), one surgeon (B.W.) and one medical oncologist (H.L.)) agreed that a complication may not be related to the radiotherapy treatment. Thirty-six (65%) of the complications were retained after exclusion of those that were deemed unrelated to radiotherapy treatment.

Of the 36 retained complications, each complication was evaluated for the grading criteria available in the data registries (Clavien-Dindo or CTCAE), each having between two and five severity grades, resulting in a total of 128 unique items evaluated in the Delphi process.

### Delphi method

The Delphi study was conducted from October 2023 until June 2024. The Delphi methodology is a structured, iterative process to achieve consensus among experts through anonymized, multi-round surveys. Participant were professionals with at least one year of (self-reported) experience in the field of EC treatment, including: surgeons, radiation oncologists, medical oncologists, gastroenterologists, physiotherapists, dietitians, and specialized nurses. Experts were recruited via the Dutch Upper GI Cancer Group (DUCG), the MODELS consortium (clinicaltrials.gov: NCT06366828) and the PROTECT consortium (clinicaltrials.gov: NCT05055648), as well as through personal networks and the corresponding authors of relevant research projects. Participants could invite up to five additional members to expand representation.

Each participant scored each grading, i.e. the Common Terminology Criteria for Adverse Events (CTCAE) version 5.0 or the Clavien-Dindo grading, for every complication [15], [16]. In the first round, participants were asked to weight each complication, with a score between 0–100, based on their perceived impact on the patient [17]. In the second round, participants received aggregated feedback on their answers from the first round, i.e. median weights and interquartile ranges of the other participants.

The Delphi process ended when consensus and agreement was reached for all complications or when responses showed no significant changes between rounds with the Wilcoxon matched-pairs signed-ranks test [18]. Based on the review on Delphi consensus by von der Gracht [18], which suggested that an interquartile range (IQR) of ≤ 2 on a 10-point scale indicates consensus among participants, our study defined consensus on a complications as an IQR of ≤ 20 on a 100-point scale. Additionally, to assure that different specialties agree on the final score, inter-group agreement was evaluated and defined as ‘no significant differences in median scores’ (Kruskal-Wallis test, p > 0.05). The disciplines were grouped into surgeons, radiation oncologists, medical oncologists/gastroenterologists and the additional care group, which included physiotherapists, dietitians, and specialized nurses. If significant differences between the different disciplines remained in the final Delphi round, a post-hoc pairwise comparison was performed using Dunn’s test, with a Bonferroni correction applied.

The final weight for each complication and each associated grading was the overall median score across experts.

### CSS and $CSS_{simplified}$ calculation

The CSS was calculated according to the same principle as the CCI [10], i.e. scaling the final score between 0–100 by taking the root and dividing by two, and scoring different levels of impact using an operation risk index approach [19]. This approach multiplies values rather than summing them, giving greater importance to complications with higher weights compared to those that are less severe.

The CSS was calculated as per equation 1:

(1) $$\begin{array}{c} \mathrm{CSS}=\frac{1}{2}\sqrt{\sum_{n=1}^{N}I_{n}{\left(S_{n}\right)}^{2}} \end{array}$$

In this equation, **I** is set to 1 if the patient has a specific complication and zero if not, **N** indicates all possible complications scored in the CSS, and **S** is the median expert score for that complication and grading.

Due to the large number of complications and gradings in the full CSS, a simplified version ($\mathrm{CS}S_{\mathrm{simplified}}$) was developed. This version uses the median value of separate gradings (Clavien-Dindo, CTCAE or miscellaneous grading) instead of assigning a weight to every complication and grading combination.

The $\mathrm{CS}S_{\mathrm{simplified}}$ was calculated as per equation 2:

(2) $$\begin{array}{c} \mathrm{CS}S_{\mathrm{simplified}}=\frac{1}{2}\sqrt{\sum_{n=1}^{N}I_{n}{\left(G_{n}\right)}^{2}} \end{array}$$

In this equation, **G** is the median expert score for the grading that got assigned to the occurred complication.

### Clinical associations of the CSS and $CSS_{simplified}$

The associations between the CSS and the $\mathrm{CS}S_{\mathrm{simplified}}$ and various clinical endpoints were assessed using two datasets. The first is the MODELS dataset, a multicenter registry of esophageal cancer patients treated with nCRT (CROSS regimen). While the broader dataset includes patients managed with or without surgery diagnosed between 2015 and 2021, the current analysis was restricted to patients who underwent an esophagectomy at a participating consortium center, and had complete data. The second dataset is the POCOP subset of this cohort, which contains additional quality-of-life (QOL) questionnaires [20]. Full inclusion and exclusion criteria are detailed in the supplementary materials.

The following hypotheses were tested to evaluate the clinical associations of the CSS and $\mathrm{CS}S_{\mathrm{simplified}}$. We first added the CSS in a univariable model and then used a forward selection procedure to add potential confounders. The confounders, which included patient (gender, age, WHO status, lung and heart comorbidities, and diabetes) and tumor (cN, cT, and histology) characteristics were retained if their p-value was less than 0.2. The final set of confounders selected for the CSS model was also applied to the $\mathrm{CS}S_{\mathrm{simplified}}$.

1. A higher CSS and $\mathrm{CS}S_{\mathrm{simplified}}$ at 1 year after nCRT and surgery are significantly associated with reduced one-year survival rates, using a multivariable logistic regression model.
2. A higher CSS and $\mathrm{CS}S_{\mathrm{simplified}}$ at discharge are significantly associated with a longer postoperative hospital and ICU length of stay, using multivariable linear regression.
3. The CSS and $\mathrm{CS}S_{\mathrm{simplified}}$ are significantly associated, using multivariable linear regression, with the patient-reported QoL, measured via the European Organization for Research and Treatment of Cancer (EORTC) QLQ-C30 summary score obtained within two months post-surgery [21]. The CSS and $\mathrm{CS}S_{\mathrm{simplified}}$ are measured at the day of the questionnaire. The baseline QLQ-C30 score is also added as a possible confounder.

All analysis were performed in R version 4.3.1 and Python version 3.10.4.

## Results

**Fig. 1:**
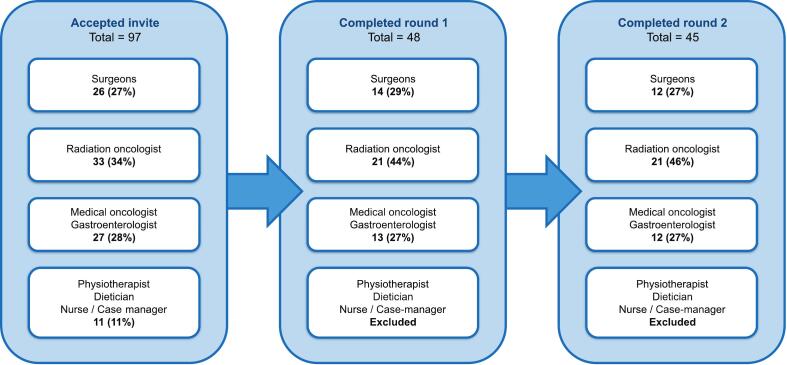
**Expert details** for every round of the Delphi study.

The participants in this Delphi study were experts in EC care, with a median experience of over 10 years. In total, forty-five experts completed the entire Delphi procedure (Fig. 1). After the first round, the additional care group was excluded as this group indicated that they lacked clinical exposure to these specific complications. Geographically, participants were distributed across nine countries: the Netherlands (32), the United Kingdom (3) Switzerland (3), Denmark (2), Sweden (1), Germany (1), Belgium (1), Czech Republic (1), and Norway (1). All professionals were (self-reported) actively engaged in care for EC-patients at the time of the study.

**Fig. 2:**
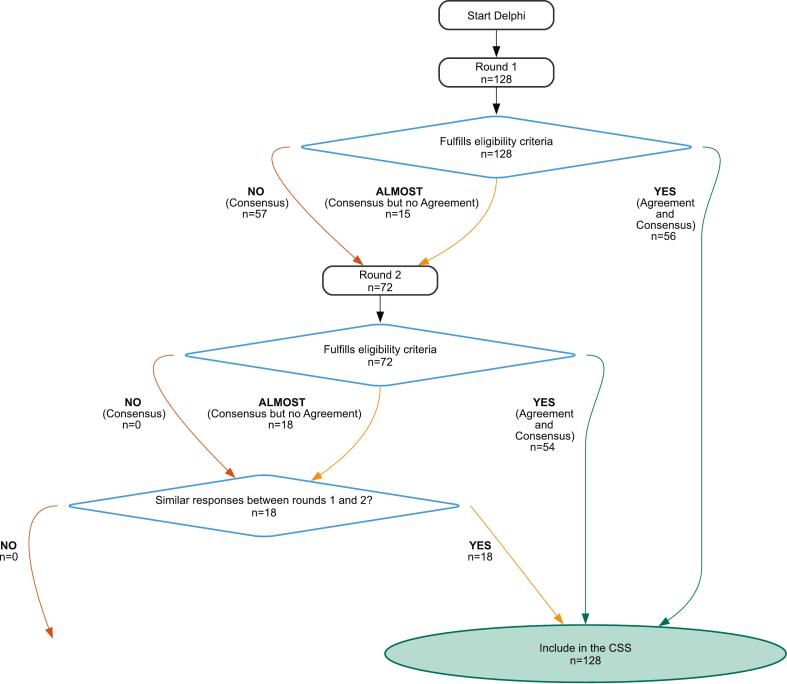
**Flowchart of group consensus** agreement between expert groups and response similarity between different Delphi rounds.

Fig. 2 shows the consensus process for the 128 unique items. The Delphi process concluded after two rounds, with consensus achieved for all items. However, eighteen items (14%) showed significant differences in assigned score between the disciplines. Medical oncologists/gastroenterologists assigned significantly higher burdens to the complications compared to surgeons (in 8 complications: median burden 34 vs 27) and radiation oncologists (in 4 complications: median burden 47 vs 40). In 4 complications, the radiation oncologists gave significantly higher scores compared to the surgeons (median burden 94 vs 84). As all items reached consensus in the complete group, answers remained unchanged between rounds, and the difference in burden between expert specialisms was minimal, all items were assigned a final weight based on the median score of all experts (Table 1).

**Table 1:** Weights for the Complication sum score*.*

	**Miscellaneous complications**			
**Title**	**Type**	**Final Weight**	**IQR**≤**20**	**Subgroup agreement**
Diabetes mellitus	Without complications	32	**✓**	**✓**
Coronary revascularization	PCI without stent	50	**✓**	**✓**
Coronary revascularization	PCI with stent	63	**✓**	**✓**
Valve disease	CTCAE Grade 3 or Grade 4	69	**✓**	**✓**
Diabetes mellitus	Withcomplications	70	**✓**	**✓**
Coronary revascularization	Coronary artery bypass grafting	82.5	**✓**	**✓**
Death from toxicity		100	**✓**	**✓**
Death from post-operative complication		100	**✓**	**✓**

	**CTCAE scored complications**			
Atrial fibrillation*	Symptomatic (Grade 2 or grade 3)	39	**✓**	**✓**
Angina pectoris	Symptomatic (Grade 2 or grade 3)	50	**✓**	**✓**
(Radiation) Pneumonia*	Symptomatic (Grade 2 or grade 3)	50	**✓**	
Congestive heart failure*	Symptomatic (Grade 2 or grade 3)	59	**✓**	**✓**
Oesophageal fistula or perforation*	Symptomatic (Grade 2 or grade 3)	62	**✓**	**✓**
Myocardial infarction*	Symptomatic (Grade 3)	64.5	**✓**	**✓**
Atrial fibrillation*	Life-threatening (Grade 4)	80	**✓**	**✓**
(Radiation) Pneumonia*	Life-threatening (Grade 4)	86	**✓**	**✓**
Congestive heart failure*	Life-threatening (Grade 4)	88	**✓**	**✓**
Myocardial infarction*	Life-threatening (Grade 4)	90	**✓**	**✓**
Oesophageal fistula or perforation*	Life-threatening (Grade 4)	95	**✓**	**✓**

	**Clavien Dindo scored complications**			
Wound infection	Grade 2	15	**✓**	
Pneumonia	Grade 2	20	**✓**	**✓**
Deep venous thrombosis	Grade 2	22	**✓**	**✓**
Thoracic wound dehiscence	Grade 2	25	**✓**	**✓**
Chyle leak	Grade 2	25	**✓**	
Postoperative bleeding	Grade 2	26	**✓**	**✓**
Liver dysfunction	Grade 2	27	**✓**	**✓**
Wound infection	Grade 3a	30	**✓**	
Pericarditis	Grade 2	30	**✓**	
Acute aspiration	Grade 2	30	**✓**	
Anastomotic leakage	Grade 2	30	**✓**	**✓**
Arrhythmia	Grade 2	30	**✓**	**✓**
Pleural effusion	Grade 3a	35	**✓**	
Tracheobronchial injury	Grade 2	38	**✓**	
Congestive heart failure	Grade 2	40	**✓**	**✓**
Atelectasis	Grade 3a	40	**✓**	**✓**
Pulmonary embolus	Grade 2	40	**✓**	**✓**
Deep venous thrombosis	Grade 3a	40	**✓**	**✓**
Pneumonia	Grade 3a	41	**✓**	**✓**
Generalized sepsis	Grade 2	42	**✓**	**✓**
Persistent air leak	Grade 3a	43	**✓**	
Thoracic wound dehiscence	Grade 3a	45	**✓**	**✓**
Acute aspiration	Grade 3a	50	**✓**	**✓**
Intrathoracic/intra-abdominal abscess	Grade 3a	50	**✓**	**✓**
Pericarditis	Grade 3a	50	**✓**	
Arrhythmia	Grade 3a	50	**✓**	
Wound infection	Grade3b	50	**✓**	
Pleural effusion	Grade 3b	50	**✓**	**✓**
Postoperative bleeding	Grade 3a	50	**✓**	**✓**
Conduit necrosis/failure	Grade 2	50	**✓**	**✓**
Anastomotic leakage	Grade 3a	50	**✓**	**✓**
Chyle leak	Grade 3a	50	**✓**	**✓**
Liver dysfunction	Grade 3a	50	**✓**	**✓**
Thoracic wound dehiscence	Grade 3b	55	**✓**	**✓**
Congestive heart failure	Grade 3a	55	**✓**	**✓**
Pulmonary embolus	Grade 3a	55	**✓**	**✓**
Deep venous thrombosis	Grade 3b	56	**✓**	**✓**
Atelectasis	Grade 3b	57	**✓**	**✓**
Pneumonia	Grade 3b	58	**✓**	**✓**
Acute Respiratory Distress Syndrome	Grade 3a	60	**✓**	**✓**
Conduit necrosis/failure	Grade 3a	60	**✓**	**✓**
Tracheobronchial injury	Grade 3a	60	**✓**	
Chyle leak	Grade 3b	60	**✓**	**✓**
Persistent air leak	Grade 3b	60.5	**✓**	**✓**
Generalized sepsis	Grade 3a	61	**✓**	**✓**
Pericarditis	Grade 3b	62	**✓**	**✓**
Acute aspiration	Grade 3b	62	**✓**	**✓**
Postoperative bleeding	Grade 3b	64	**✓**	**✓**
Intrathoracic/intra-abdominal abscess	Grade 3b	65	**✓**	**✓**
Liver dysfunction	Grade 3b	65	**✓**	**✓**
Arrhythmia	Grade 3b	66	**✓**	**✓**
Pulmonary embolus	Grade 3b	66.5	**✓**	**✓**
Anastomotic leakage	Grade 3b	67.5	**✓**	**✓**
Congestive heart failure	Grade 3b	70	**✓**	
Tracheobronchial injury	Grade 3b	72.5	**✓**	**✓**
Acute Respiratory Distress Syndrome	Grade 3b	73	**✓**	**✓**
Generalized sepsis	Grade 3b	74	**✓**	**✓**
Wound infection	Grade 4a	75	**✓**	
Pleural effusion	Grade 4a	76	**✓**	**✓**
Conduit necrosis/failure	Grade 3b	80	**✓**	**✓**
Thoracic wound dehiscence	Grade 4a	80	**✓**	**✓**
Atelectasis	Grade 4a	80	**✓**	**✓**
Persistent air leak	Grade 4a	80	**✓**	**✓**
Pneumonia	Grade 4a	80	**✓**	**✓**
Deep venous thrombosis	Grade 4a	80	**✓**	**✓**
Pericarditis	Grade 4a	81	**✓**	**✓**
Acute aspiration	Grade 4a	81	**✓**	**✓**
Postoperative bleeding	Grade 4a	82.5	**✓**	**✓**
Liver dysfunction	Grade 4a	83	**✓**	**✓**
Chyle leak	Grade 4a	83	**✓**	**✓**
Intrathoracic/intra-abdominal abscess	Grade 4a	83.5	**✓**	**✓**
Arrhythmia	Grade 4a	85	**✓**	**✓**
Stroke	Grade 4a	85	**✓**	**✓**
Acute Respiratory Distress Syndrome	Grade 4a	85	**✓**	**✓**
Anastomotic leakage	Grade 4a	85.5	**✓**	**✓**
Congestive heart failure	Grade 4a	86	**✓**	**✓**
Respiratory failure	Grade 4a	86	**✓**	**✓**
Tracheobronchial injury	Grade 4a	86.5	**✓**	**✓**
Pulmonary embolus	Grade 4a	87.5	**✓**	**✓**
Myocardial infarction	Grade 4a	88	**✓**	**✓**
Generalized sepsis	Grade 4a	89	**✓**	**✓**
Conduit necrosis/failure	Grade 4a	89.5	**✓**	**✓**
Wound infection	Grade 4b	90	**✓**	
Deep venous thrombosis	Grade 4b	90	**✓**	**✓**
Pleural effusion	Grade 4b	93	**✓**	**✓**
Atelectasis	Grade 4b	94	**✓**	
Thoracic wound dehiscence	Grade 4b	94	**✓**	**✓**
Persistent air leak	Grade 4b	94	**✓**	**✓**
Acute aspiration	Grade 4b	94	**✓**	**✓**
Pneumonia	Grade 4b	94.5	**✓**	**✓**
Arrhythmia	Grade 4b	95	**✓**	
Pericarditis	Grade 4b	95	**✓**	**✓**
Cardiac arrest	Grade 4a	95	**✓**	**✓**
Chyle leak	Grade 4b	95	**✓**	**✓**
Intrathoracic/intra-abdominal abscess	Grade 4b	95	**✓**	**✓**
Postoperative bleeding	Grade 4b	95	**✓**	**✓**
Pulmonary embolus	Grade 4b	95.5	**✓**	**✓**
Liver dysfunction	Grade 4b	95.5	**✓**	**✓**
Respiratory failure	Grade 4b	95.5	**✓**	**✓**
Acute Respiratory Distress Syndrome	Grade 4b	96.5	**✓**	**✓**
Congestive heart failure	Grade 4b	97	**✓**	**✓**
Stroke	Grade 4b	97	**✓**	**✓**
Myocardial infarction	Grade 4b	97	**✓**	**✓**
Conduit necrosis/failure	Grade 4b	97	**✓**	**✓**
Generalized sepsis	Grade 4b	97.5	**✓**	**✓**
Anastomotic leakage	Grade 4b	97.5	**✓**	**✓**
Tracheobronchial injury	Grade 4b	97.5	**✓**	**✓**
Cardiac arrest	Grade 4b	100	**✓**	**✓**
Multiple organ dysfunction syndrome	Grade 4b	100	**✓**	**✓**

* not accounted for in the 30 day postoperative period to prevent overlap of complications in the Netherlands Cancer Registry and the Dutch Upper GI Cancer Audit.

**Fig. 3:**
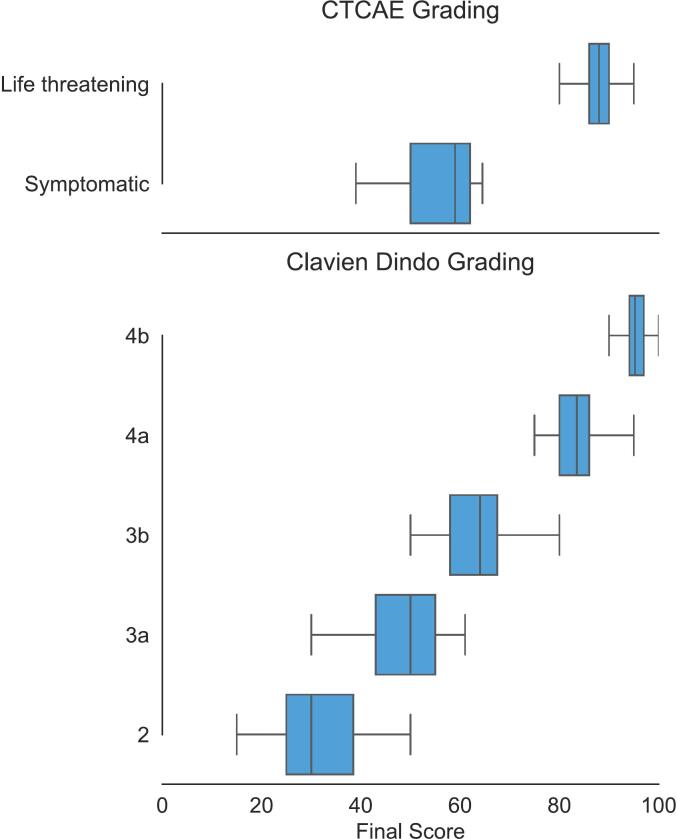
**Distribution of the different complication weights** across complications with the same CTCAE or Clavien-Dindo gradings.

**Fig. 4:**
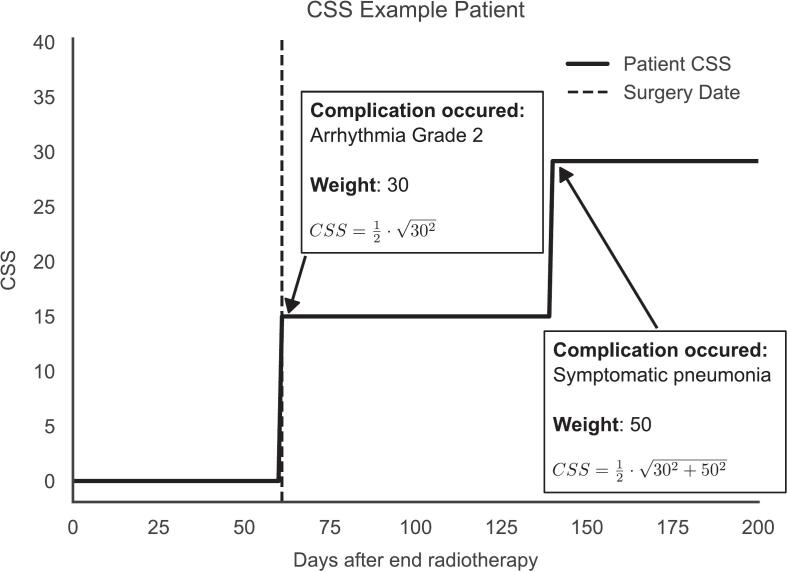
**Example of the complication sum score (CSS).** At 75 days after radiotherapy, the patient underwent surgery. In the postoperative period, the patient experienced a Clavien-Dindo grade 2 dysrhythmia, which was assigned a weight of 30 in the Delphi process. At 140 days after radiotherapy, the patient experienced a symptomatic pneumonia, which was assigned a weight of 50. This led to the patient’s CSS of 29.

Higher-grade complications had narrower interquartile ranges (IQRs) among experts, as illustrated in Fig. 3. Fig. 4 provides an example of how to calculate the CSS for a patient using the Delphi-assigned weights. In this example, the patient underwent surgery 75 days post-radiotherapy and subsequently developed two notable complications: a postoperative Clavien-Dindo grade II dysrhythmia (Delphi weight = 30; which led to a CSS of 15) and, at 140 days post-radiotherapy, symptomatic pneumonia (Delphi weight = 50). Together, these events yielded a cumulative CSS of 29. The final weights for the $\mathrm{CS}S_{\mathrm{simplified}}$ are provided in the supplementary data.

**Fig. 5:**
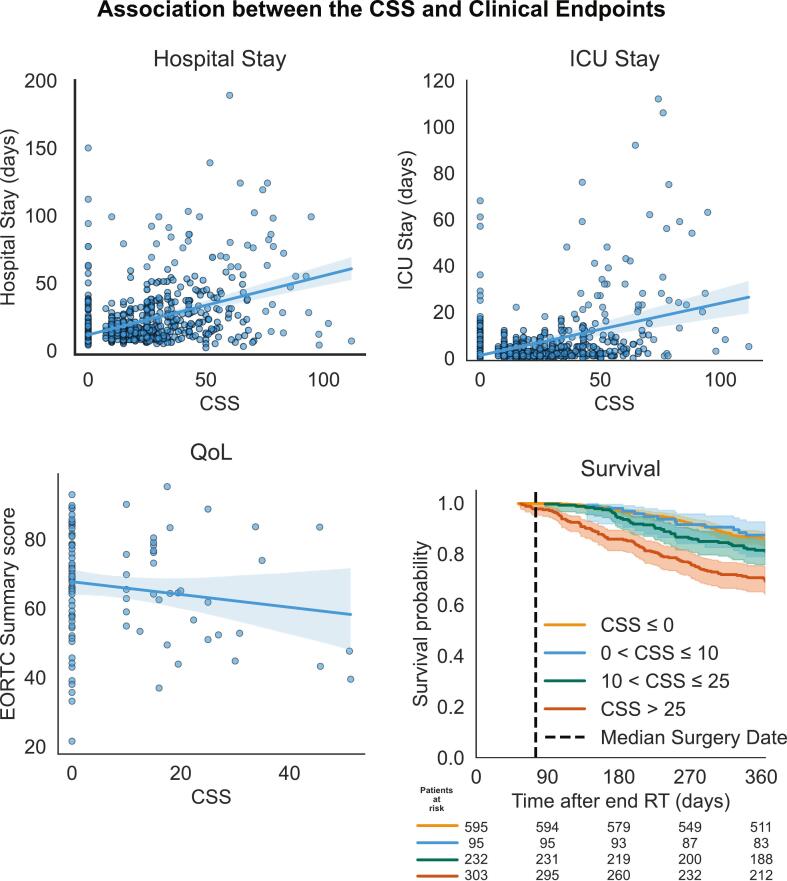
**Association between the complication sum score (CSS) and various clinical endpoints.** Quality of life (QoL) is measured via the European Organization for Research and Treatment of Cancer (EORTC) QLQ-C30 summary score obtained within two months post-surgery. The survival curves are stratified by the CSS quartiles. For visualization purposes, one outlier was excluded from the hospital stay figure (hospital stay of 380 days with a CSS of 0) and two were excluded from the intensive care unit (ICU) stay figure (ICU stay of 183 and 155 days with CSS scores of 60 and 0 respectively).

Baseline characteristics of the MODELS cohort, which was used to evaluate the associations between CSS and several clinical endpoints, are shown in the supplementary data. For this analysis, 1225 patients were included of which 993 (81%) were alive one year after completing nCRT. During the forward selection, locking the CSS as predictor, n-stage (coefficient = 0.06 [0.02–0.11], p < 0.01), WHO status ≥ 1 (coefficient = 0.04 [0.00–0.08], p = 0.08), and the female sex (coefficient = -0.04 [-0.09–0.02], p = 0.16), were found to associate with survival. In the resulting multivariable model both the CSS as well as the $\mathrm{CS}S_{\mathrm{simplified}}$ were significantly associated with one-year survival, with an odds ratio (OR) of 1.005 [1.004–1.006] and 1.004 [1.003–1.005] (p < 0.01 for both) respectively. For illustrative purposes, a survival curve stratified by CSS quartiles is presented in Fig. 5, showing that patients in the highest CSS quartile (>25) showed a notably worse survival.

The median postoperative hospital stay was 11 days, and the median ICU stay 1 day. Both the CSS as well as the $\mathrm{CS}S_{\mathrm{simplified}}$ was a significant predictor of prolonged hospital stay (both p < 0.01), even after adjusting for age (coefficient = 0.10 [-0.03 – 0.23], p = 0.13), which was identified as a confounder via forward selection. Specifically, each one-point increase in CSS or in $\mathrm{CS}S_{\mathrm{simplified}}$ was associated with a 0.43 day [0.38 – 0.48] and 0.42 day [0.37 – 0.47] increase in hospital stay respectively. Regarding ICU-stay, no confounders were identified. Both scores were significantly associated with longer ICU stay, with each additional CSS or $\mathrm{CS}S_{\mathrm{simplified}}$ point corresponding to a 0.23-day [0.19 – 0.26] or 0.21-day [0.18 – 0.24] increase respectively (both p < 0.01).

A total of 98 patients (8%) completed the EORTC QLQ-C30 questionnaire within two months post-surgery. The median decline in summary score was 18 points (scale: 0–100, with 100 being best possible QoL). During forward selection, the female sex (coefficient = -7.44 [-15.45 – 0.56], p = 0.07), WHO status 1+ (coefficient = -5.08 [-11.47 – 1.30], p = 0.12), and a lung comorbidity (coefficient = -8.17 [-19.32 – 2.97], p = 0.15) were found to associate with the QoL. Both CSS and $\mathrm{CS}S_{\mathrm{simplified}}$ scores were associated with worse patient-reported QoL. Specifically, each one-point increase in CSS or $\mathrm{CS}S_{\mathrm{simplified}}$ corresponded to a 0.24 points [-0.01 – 0.49] (p = 0.06) and 0.23 points [-0.01–0.46] (p = 0.06) decrease in QoL respectively. Fig. 5 presents scatterplots along with univariable associations for all clinical endpoints.

## Discussion

This study developed a complication score, i.e. CSS, that quantifies the treatment burden in EC patients undergoing trimodality treatment, for all complications that could potentially be related to radiotherapy. Forty-five experienced health care professionals from various disciplines participated in the Delphi study, through which the CSS was developed. The CSS demonstrated significant associations with hospital stay, ICU stay, and survival in a multicenter, multidisciplinary dataset of EC patient treated with a trimodality treatment.

A higher CSS was significantly associated with prolonged hospital stay, aligning with previous findings on the impact of complications on hospitalization stay [22]. Additionally, as anticipated based on earlier reports linking intraoperative complications to extended ICU stays [23], an increasing CSS was also significantly associated with prolonged ICU stay. Specifically, each point increase in CSS corresponded to a 0.44-day extension in hospital stay and a 0.22-day increase in ICU stay, indicating a substantial, clinically relevant, association. Furthermore, the observed association between the CSS and survival in the present study supports findings from a recent meta-analysis showing that postoperative complications are linked to worse long-term survival [24]. Our results show a clinically relevant decline in patients in the fourth CSS quartile, i.e. a CSS exceeding 25 (Fig. 5).

Finally, a higher CSS trended towards a significant association with worse post-surgical QoL, albeit barely, with only a 0.25-point decrease per CSS step. The literature on the relationship between QoL and complications is mixed: Downey et al. [25] observed a significant association between complications and QoL in adults undergoing major abdominal surgery, whereas Jezerskyte et al. [26] found no such link in patients who underwent an esophagectomy. However, there is a substantial risk of bias in these QoL analysis. In our cohort, QoL questionnaire data was not available in patients with CSS scores above 51 (Fig. 5), indicating a potential bias. Individuals experiencing more severe complications seem unable to respond; this likely underestimates the true impact of severe complications on QoL.

Consensus was achieved on all complications after just two rounds of the Delphi process. The experts agreed on the score for 86% of complications. For the remaining 14%, surgeons consistently rated the complication burden lowest, whereas medical oncologists/gastroenterologists assigned the highest scores; radiation oncologists’ assessments fell between these. This divergence underscores the necessity of incorporating broad, multidisciplinary expert input when developing a complication sum score. Earlier methods, such as the CCI, rely solely on input from surgical teams, which may lead to an underestimation of the scores.

This Delphi study was initiated to gain consensus on a single score reflecting the burden of complications in which radiotherapy may play a role in a cohort of EC patients who received nCRT. Although many of the complications occurred in the postoperative period, the expert panel considered them potentially related to the nCRT. While surgery may be the immediate trigger, the radiation dose to OARs significantly increases the likelihood of these events. For example, pneumonia is strongly associated with the radiation dose to the lungs [27], atrial fibrillation with the dose to the pulmonary vein [28], and anastomotic leakage with the dose to the gastric fundus [29], [30].

Although the ESOPEC trial supports perioperative chemotherapy (FLOT) for adenocarcinoma [31], nCRT remains the standard for squamous cell carcinoma, organ preservation strategies, and patients unfit for triplet chemotherapy. Therefore, optimizing radiotherapy planning remains essential.

The CSS facilitates this in two separate ways. First, it can serve as a single, validated composite endpoint for NTCP modeling [32], aggregating (rare) complications into a cumulative burden to overcome the statistical limitations of modeling individual events. Second, the CSS weighting factors can be integrated into multi-objective end point directed treatment planning [7]. In this framework, when utilizing separate established NTCP models, such as those by Thomas et al. or Berbee et al. for pneumonia or mortality [3], [33], the Delphi-derived weights act as priority coefficients. This allows clinicians to quantitatively weight the importance of the risks, guiding the planning system to prioritize sparing organs that contribute to the most clinically burdensome complications. By either predicting the CSS directly or using its weights to balance multiple NTCP models during end point directed optimization, radiotherapy can be more precisely tailored to minimize the overall complication burden.

In the current study, we also developed the $\mathrm{CS}S_{\mathrm{simplified}},$ a more practical alternative that assigns scores based solely on complication grades. There were no major differences in the clinical associations between the CSS and $\mathrm{CS}S_{\mathrm{simplified}}$. However, experts assigned distinct weights to complications within the same grade, indicating differences in perceived impact to the patient. This variation was particularly notable for low-grade complications, as illustrated in Fig. 3, where the IQR was widest. Since these low-grade complications are most frequently observed, we recommend implementation of the more detailed CSS over the $\mathrm{CS}S_{\mathrm{simplified}}$ to capture the nuanced burden of complications.

Limitations of our study include the exclusive involvement of Western healthcare professionals, the absence of direct patient input, and the assessment of clinical associations solely within Dutch centers and among patients who underwent surgery. Given that the characteristics of EC patients, such as the tumor histology, can vary across populations [34], the applicability of the CSS to non-Western settings remains uncertain. Future research should prioritize validating the CSS in non-Western settings to ensure its generalizability. Furthermore, the CSS has not been validated in patients who do not undergo esophagectomy. For patients not receiving surgery, validating the non-surgical complication weightings remains a future priority to ensure the tool can accurately predict and mitigate adverse events in non-surgical settings. Finally, direct patient input was explored via a focus group but deemed unfeasible due to the difficulty of patients grading complications they had not personally experienced.

## Conclusion

In summary, the CSS offers a comprehensive metric to quantify and summarize complications in EC patients that undergo neoadjuvant chemoradiation followed by surgery. Given its significant associations with hospital stay, ICU stay, and 1-year survival, the CSS may serve as a composite endpoint for NTCP modeling and as such guide treatment planning to minimize the overall complication burden and enhance patient outcomes.

## Data Availability Statement for this Work

Research data are stored in an institutional repository and will be shared upon reasonable request to the corresponding author.

## Funding statement

This study was financially supported by the Dutch Cancer Society (14303). No external parties regarding funding were involved in the study design, analysis or writing.

## Declaration of Generative AI and AI-assisted technologies in the writing process

During the preparation of this work the author(s) used ChatGPT in order to proofread. After using this tool/service, the author(s) reviewed and edited the content as needed and take(s) full responsibility for the content of the publication.

## CRediT authorship contribution statement

**M.L. Frederiks:** Conceptualization, Methodology, Formal analysis, Investigation, Data curation, Writing – original draft. **M. Berbée:** Conceptualization, Methodology, Resources, Data curation, Writing – review & editing. **B.P.L. Wijnhoven:** Conceptualization, Resources, Writing – review & editing. **E. Schuit:** Methodology, Validation, Writing – review & editing. **P.S.N. van Rossum:** Data curation, Resources, Writing – review & editing. **G.J. Meijer:** Data curation, Resources, Writing – review & editing. **S. Mook:** Data curation, Resources, Writing – review & editing. **J.J. Nuyttens:** Data curation, Resources, Writing – review & editing. **H. Rütten:** Data curation, Resources, Writing – review & editing. **M.D. den Hartogh:** Data curation, Resources, Writing – review & editing. **B. van Etten:** Data curation, Resources, Writing – review & editing. **J.A. Langendijk:** Conceptualization, Methodology, Resources, Writing – review & editing, Supervision. **H.W.M. van Laarhoven:** Conceptualization, Methodology, Resources, Writing – review & editing. **C.T. Muijs:** Methodology, Data curation, Conceptualization, Validation, Writing – original draft, Writing – review & editing, Supervision, Funding acquisition. **M. Berbee:** . **H.P. van der Laan:** . **A. van der Schaaf:** . **J.J.M.E. Nuyttens:** . **H. Rutten:** . **Y.L.B. Klaver:** . **R.H.A. Verhoeven:** . **J.J. de Haan:** . **W. Kleder:** . **M.I. van Berge Henegouwen:** . **Z. van Kesteren:** . **B. Mostert:** . **W. Schillemans:** . **M. Sosef:** . **F.A.R.M. Warmerdam:** . **R. Canters:** . **J.P. Ruurda:** . **N. Haj Mohammad:** . **B. Klarenbeek:** . **H. Westdorp:** .

## Declaration of Competing Interest

The authors declare the following financial interests/personal relationships which may be considered as potential competing interests: **.W.** received research grant form BMS, consulting fees from BMS and Medtronic. **J.A.L.** reports department research contracts with IBA, RaySearch, Siemens, Elekta, and Mirada, has received grants from the Dutch Cancer Society and European Union (EU), has received consulting fees as a member of Global Scientific Advisory Board of IBA (payments made to University Medical Center Groningen (UMCG) Research BV); payment or honoraria for presentations for local scientific meetings for IBA (payments made to UMCG Research BV); is also a member of RayCare International Advisory Board of RaySearch (no payments), is chair of the Safety Monitoring Committee of the UPGRADE-trial and reports at the University Medical Center Nijmegen, is a member of the Global Advisory Committee of IBA, and is a chair of the Netherlands Society for Radiation Oncology (NVRO) (unpaid). **C.M.** reports department research contracts with IBA, RaySearch, Siemens, Elekta, and Mirada, and has received grants from the Dutch Cancer Society and EU. **H.L.** declares research funding and/or medication supply: Amphera, Anocca, Astellas, AstraZeneca, Beigene, Boehringer, Daiichy-Sankyo, Dragonfly, MSD, Myeloid, ORCA, Servier. Consultant/advisory role: Auristone, Incyte, Merck, Myeloid, Servier. Speaker role: Astellas, AstraZeneca, Beigene, Benecke, BMS, Daiichy-Sankyo, JAAP, Medtalks, Novartis, Springer, Travel Congress Management B.V **MIvanBH** declares consultancies for Johnson and Johnson, Stryker, Boston Scientific, Intuitive and Medtronic. All other authors declare no potential conflicts of interest. No external parties regarding funding were involved in the study design, analysis, or writing..
