# Supplementary material for: The complication sum score: A Delphi-based approach to summarize treatment related complications for esophageal cancer
Source: Clin Transl Radiat Oncol. 2026 Mar 15;59:101146. doi: 10.1016/j.ctro.2026.101146 (PMC13087729; doi:10.1016/j.ctro.2026.101146)
Supplement: Supplementary Data 1 [file mmc1.docx]

Appendix A

**Delphi study details**

The experts of the study participated in two Delphi rounds. Figure A1, shows an example question of the Delphi study, as given in the second round.

**Validation data details**

The MODELS dataset is a multicentre, multidisciplinary dataset. Patient data were prospectively maintained in the Netherlands Cancer Registry and the Dutch Upper GI Cancer Audit, radiotherapy data were retrospectively collected from the following hospitals: X1, X2, X3, X4, X5, and X6. Patients were treated with neoadjuvant chemo-radiotherapy according to the CROSS schedule with a planned esophagectomy.

Inclusion criteria for the dataset were:

1. Diagnosed between 2015 and 2021
2. Patients diagnosed with oesophageal cancer treated with curative intent using chemoradiotherapy according to the CROSS regimen, planned for a esophagectomy.
3. Both chemoradiotherapy and, if performed, surgery must have been performed at one of the participating consortium centres. Which included: X1, X2, X3, X4, X5, X6, X7, X8, X9 and X10.

The POCOP dataset is a subset of the MODELS dataset, including only patients with completed quality-of-life questionnaires. Patient characteristics are summarized in Table A1. Patients where excluded if no informed consent was provided.

**Details about the CSS calculation.**

The Complication Sum Score (CSS) was calculated using the following formula:

$$CSS= \frac{1}{2}\sqrt{\sum_{n=1}^{N} I_{n}\left( S_{n} \right)^{2}}$$

n this equation, **I** is set to 1 if the patient has a specific complication and 0 if not, **N** indicates all possible complications scored in the CSS, and **S** is the median expert score for that complication and grading.

For the complications recorded in the Netherlands Cancer Registry (IKNL) database the score is assigned on the exact date the complication occurred. In contrast, since the Dutch Upper GI Cancer Audit (DUCA) database only indicates that a complication is within the 30-day postoperative period without specifying the date, those complications are assigned the day of resection.

Five complications appear in both databases: pneumonia, atrial fibrillation, myocardial infarction, oesophageal leakage and heart failure. To prevent doubles and to account for differences in complication weight, these complications are not included from the IKNL database for the 30-day postoperative period.

If a patient had a unknown grading, the complication is not counted in the CSS. Moreover, if a patient experienced multiple grade 5 complications (death), one of them, the complication with the lowest $S_{n}$, was retained as grade 5. Any additional grade 5 complications were downgraded to grade IV, reflecting the principle that a patient can only have one fatal outcome.

| **Variable name** | | QOL  n= 98 | Hospital, ICU stay and survival  n=1225 |
| --- | --- | --- | --- |
| **Age** | Median | 65 | 66 |
| **Gender** | Male | 81 (83%) | 984 (80%) |
|  | Female | 17 (17%) | 241 (20%) |
| **Histology** | Adenocarcinoma | 84 (86%) | 1032 (84%) |
|  | Squamous cell carcinoma | 14 (14%) | 193 (16%) |
| **cN** | cN0 | 37 (38%) | 461 (38%) |
|  | cN1 | 40 (41%) | 460 (38%) |
|  | cN2 | 20 (20%) | 272 (22%) |
|  | cN3 | 1 (1%) | 32 (3%) |
| **cT** | cT1 | 1 (1%) | 12 (1%) |
|  | cT2 | 28 (29%) | 338 (28%) |
|  | cT3 | 67 (68%) | 857 (70%) |
|  | cT4 | 2 (2%) | 18 (1%) |
| **Rt-technique** | VMAT | 87 (89%) | 855 (70%) |
|  | 3DCRT | 7 (7%) | 130 (11%) |
|  | HYBRID | 2 (2%) | 146 (12%) |
|  | IMPT | 0 (0%) | 73 (6%) |
|  | IMRT | 1 (1%) | 12 (1%) |
|  | Other | 0 (0%) | 1 (0%) |
|  | Missing | 1 (1%) | 8 (1%) |
| **Type of procedure** | Minimally invasive thorax and abdomen | 77 (79%) | 837 (68%) |
|  | Minimally invasive abdomen (including minimally invasive transhiatal) | 18 (18%) | 209 (17%) |
|  | Open procedure | 2 (2%) | 132 (11%) |
|  | Minimally invasive thorax | 1 (1%) | 47 (4%) |
| **Location of anastomosis** | Intrathoracic | 66 (67%) | 768 (63%) |
|  | Neck | 29 (30%) | 426 (35%) |
|  | Intra-abdominal | 0 (0%) | 5 (0%) |
|  | No anastomosis | 0 (0%) | 4 (0%) |
|  | Other | 0 (0%) | 2 (0%) |
|  | Unknown | 0 (0%) | 2 (0%) |
|  | Missing | 3 (3%) | 18 (1%) |
| **Type of resection** | Transthoracic esophagectomy | 91 (93%) | 1112 (91%) |
|  | Transhiatal esophagectomy | 7 (7%) | 106 (9%) |
|  | Total gastrectomy | 0 (0%) | 7 (1%) |

**Table A1**: Patient characteristics for the subsets for testing the clinical relevance of the complication sum score against quality of life (QOL), overall survival, and hospital/ICU stay. HYBRID = a combination of 3DCRT with VMAT or IMRT.

**Figure A1:** Example survey question of the second round of the Delphi study. The participant answered with a weight of 24 the first round and 40 the second round.


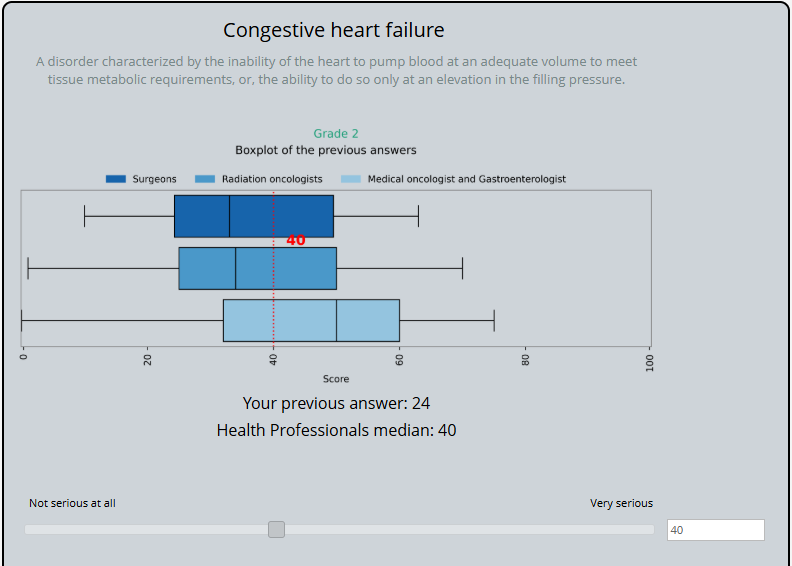


**Table A2:** *Weights for the* $ComplicationSum{Score}_{simplified}$*.*

| **Clavien Dindo grading** | | |
| --- | --- | --- |
| **Title** | **Final Weight** | |
| Grade 2 | 30 | |
| Grade 3a | 50 | |
| Grade 3b | 64 | |
| Grade 4a | 84 | |
| Grade 4b | 95 | |
| **CTCAE grading** | | |
| Grade 2/3 | 59 | |
| Grade 4 | 88 | |
| **Miscellaneous complications** | | |
| **Title** | **Type** | **Final Weight** |
| Diabetes mellitus | Without  complications | 32 |
| Coronary revascularization | PCI without stent | 50 |
| Coronary revascularization | PCI with stent | 63 |
| Valve disease | CTCAE Grade > 2 | 69 |
| Diabetes mellitus | With complications | 70 |
| Coronary revascularization | Coronary artery  bypass grafting | 82.5 |
| Death from toxicity |  | 100 |
| Death from post-operative complication |  | 100 |
